# Ezrin promotes esophageal squamous cell carcinoma progression via the Hippo signaling pathway

**DOI:** 10.1515/biol-2022-0743

**Published:** 2023-09-30

**Authors:** Lijuan Ma, Li Liu, Min Ji, Liping Su, Yaling Guan, Jinling Xiao, Hongwei Pu

**Affiliations:** State Key Laboratory of Pathogenesis, Prevention and Treatment of High Incidence Diseases in Central Asia, Department of Physiology, School of Basic Medical Sciences, Xinjiang Medical University, No. 567, Shangde North Road, Urumqi 830017, Xinjiang, China; Department of Pathology, First Affiliated Hospital, Xinjiang Medical University, No. 137, Liyushan South Road, Urumqi 830054, Xinjiang, China; Department of Pathology, School of Basic Medical Sciences, Xinjiang Medical University, No. 567, Shangde North Road, Urumqi 830017, Xinjiang, China; Department of Discipline Construction, First Affiliated Hospital, Xinjiang Medical University, No. 137, Liyushan South Road, Urumqi 830054, Xinjiang, China

**Keywords:** esophageal squamous cell carcinoma, ezrin, yes-associated protein, Hippo signaling pathway, connective tissue growth factor

## Abstract

The aim of this study was to analyze the role of Ezrin in esophageal squamous cell carcinoma (ESCC) and investigate potential therapeutic targets for ESCC by interfering with Ezrin expression. Bioinformatics analysis revealed that Ezrin expression differed significantly among patients with different clinical stage ESCC. Moreover, there was a significant correlation between Ezrin and yes-associated protein/connective tissue growth factor (YAP_1_/CTGF) levels in esophageal cancer. Sixty paraffin-embedded ESCC tissue samples were examined and Ezrin and YAP_1_/CTGF levels were determined using immunohistochemistry. The positive expression rates of Ezrin and YAP_1_/CTGF were significantly lower in adjacent tissues than in ESCC tissues. Furthermore, knockdown of Ezrin expression inhibited colony formation and reduced cell migration and invasion. Compared with control ESCC cells, protein expression levels of YAP_1_ and CTGF were significantly downregulated in cells with Ezrin knocked down. We conclude that Ezrin may be involved in ESCC progression through the Hippo signaling pathway.

## 1 Introduction

Esophageal cancer is the seventh most common cancer in the world (by incidence), and the sixth deadliest, according to mortality rates. Mortality rates for new cases of esophageal cancer are >50%, because of its highly aggressive characteristics and due to frequent diagnosis at a late stage of disease, resulting in a 5 year survival rates of approximately 12–39%. From a global perspective, the incidence rate of esophageal cancer in China ranks first in the world, and >540,000 people died of esophageal cancer in 2020 worldwide, of which almost 50% of deaths were in China. Esophageal cancer can be histologically classified into esophageal adenocarcinoma and esophageal squamous cell carcinoma (ESCC), with ESCC accounting for >90% of esophageal cancer cases in China [1,2,3,4].

Ezrin, a protein belonging to the Ezrin/radixin/moesin family, is abnormally expressed in various tumors, including ESCC [5,6]. Ezrin has an important role in maintenance of protein structural stability and integrity, and can promote cancer occurrence and progression [7,8]. High levels of Ezrin gene expression are linked to cell proliferation, migration, invasion, and tumor differentiation. Enhancers are elements that regulate gene expression and non-small cell lung cancer invasion and metastasis can be reduced by knocking the Ezrin enhancer down or out [9]. Ezrin promotes artery epithelial cell proliferation, migration, and angiogenesis through Hippo–yes-associated protein (YAP) signaling [10]. The Hippo signaling pathway plays a unique regulatory role in biological processes, such as organ size and tissue regeneration, and is strongly implicated in tumor development and progression [11,12]. YAP is a key effector in Hippo pathway signal transduction, and is overexpressed in the nuclei of human colorectal cancer, esophageal cancer, ovarian cancer, hepatocellular carcinoma, and lung cancer cells [13,14]. Further, YAP can be used as a marker for clinical tumor diagnosis and prognosis [15,16]. In addition, as a direct target gene downstream of YAP, the expression of connective tissue growth factor (CTGF) is positively correlated with the activity of Hippo/YAP_1_ pathway [17]. In cancer, CTGF can promote tumor occurrence, progression, and metastasis by regulating cell proliferation, migration, and invasion. The YAP/CTGF axis is important in tumor malignant progression. For example, SREBP1 overexpression can upregulate CTGF levels through the Hippo–YAP pathway and promote thyroid cell invasion [18]. Ezrin promotes the invasion and metastasis of some tumors by upregulating YAP_1_ expression [19]. Based on these data, we speculated that the mechanism of YAP_1_ promotion of ESCC development may be related to Ezrin, which has a regulatory effect on the YAP_1_/CTGF signaling pathway. To test this hypothesis, we constructed ESCC cells with targeted Ezrin knockdown, and studied the specific molecular mechanism by which Ezrin promotes ESCC malignant progression at the cellular and molecular levels, as well as the interaction between Ezrin and YAP_1_ in ESCC occurrence and development, providing new targets for the clinical prevention or treatment of ESCC.

## 2 Materials and methods

### 2.1 Materials

#### 2.1.1 Clinical tissue samples

A total of 60 paraffin-embedded ESCC tissue samples were collected from the First Affiliated Hospital of Xinjiang Medical University between 2008 and 2018.


**Informed consent:** Informed consent was obtained from all individuals included in this study.
**Ethical approval:** Research related to human participants complied with all relevant national regulations and institutional policies, and was conducted in accordance with the tenets of the Helsinki Declaration. The study was approved by the Ethics Committee of The First Affiliated Hospital of Xinjiang Medical University (K201909-03).

### 2.2 Methods

#### 2.2.1 Online database analysis

Analysis of Ezrin expression in ESCC and normal esophageal tissue samples was conducted using data from the UALCAN database. Data from the GEPIA and UALCAN databases were used to analyze Ezrin expression in different clinical stages of esophageal cancer, and correlation between Ezrin and YAP_1_ expression levels was assessed using data from GEPIA. Additionally, a network of protein–protein interactions involving Ezrin was constructed using STRING.

#### 2.2.2 Cell culture

Human ESCC cell lines (KYSE30, KYSE150, and KYSE450) and a human esophageal epithelial cell line (SHEE) were obtained from the Shanghai Cell Bank (Shanghai, China). A lentivirus vector for Ezrin gene knockdown and a negative control vector were purchased from Shanghai Genechem Co., Ltd. The specific sequences were as follows: EZR-RNAi (89218-1): 5′-CGTGGGATGCTCAAAGATAAT-3′; EZR-RNAi (89219-1): 5′-GGGCAACCATGAGTTGTATAT-3′; EZR-RNAi (89220-1): 5′-GAGGACC CACAATGACATCAT-3′; Negative control: 5′-TTCTCCGAACGTGTCACGT-3′. Cells were cultured in DMEM (Gibco Co., USA), supplemented with 10% fetal bovine serum (BI Co., Israel), and penicillin/streptomycin (1%) (Gibco Co., USA).

#### 2.2.3 Cell transfection

Cells in logarithmic growth were seeded in 24-well culture plates at 3 × 10^6^ cells per well. Cultures were continued for 72 h to observe transfection efficiency. Transfected cells were observed under an inverted fluorescence microscope, then screened by flow cytometry and western blotting to evaluate target protein knockout efficiency.

#### 2.2.4 Wound-healing assay

Wound-healing assays were conducted to evaluate cell migration rates. Cells in logarithmic growth phase were seeded into six-well plates at 5 × 10^5^ cells/mL and cultured to 90% confluence. A 1 mL pipette tip was used to draw a straight line wound in the adherent cells and the scratch subsequently observed under a microscope to obtain images after 0 and 48 h. Cell migration rates were evaluated based on the average scratch width ratio.

#### 2.2.5 Colony formation assay

Cells were inoculated in six-well plates (500 cells/well) and cultured for 7–14 days. After the cells formed clonal groups, they were fixed with 4% histiocytic cell fixation solution at room temperature for 20 min, then 1 mL of 0.1% crystal purple (McLean, China) was added to each well for 2 min, followed by washing with PBS until the staining solution was washed away and clusters of cell clones stained purple were visible.

#### 2.2.6 Transwell assays

For migration assays, cell suspensions were added to the upper chambers of Transwell dishes and 500 μL of complete culture medium was added to each lower chamber. After 24 h culture, cells were fixed with 4% paraformaldehyde and stained with crystal violet for 15 min. For invasion assays, the procedure used was similar to that for the migration assay, except that cells in upper chambers were embedded in Matrigel (Corning Incorporated, Corning, NY, USA). Images of five randomly selected microscopic fields of fixed cells were captured and the number of cells that had migrated or invaded tallied.

#### 2.2.7 Western blotting

Cells were collected and lysed using RIPA buffer solution (Abcam Inc., ab156034). Protein content was measured using the BCA method and 30 μg protein aliquots of each sample was separated by SDS-polyacrylamide gel electrophoresis (Solarbio Company, China). Separated proteins were transferred to PVDF membranes (Merck Millipore, Billerica, MA, USA), which were blocked for 1 h and then incubated with primary antibodies specific for Ezrin (1:500) (Abcam Inc., ab4069), YAP_1_ (1:500) (Abcam Inc., ab52771), CTGF (1:700) (Proteintech, USA, 23936-1-AP), and GAPDH (1:500) (Abcam Inc., ab181602) overnight at 4°C. Subsequently, secondary antibodies were added and incubated at room temperature for 1 h. The chemiluminescence signals were generated by X-ray film. Finally, protein expression was quantified using Image J software.

#### 2.2.8 Immunohistochemical analysis

Paraffin sections were processed by xylene dewaxing, ethanol hydration, and blocking with PBS (HyClone Company, USA) containing normal goat serum. Primary antibodies against Ezrin, YAP_1_, and CTGF (1:50) were added and incubated overnight at 4°C in a wet box. Subsequently, secondary antibody and DAB (Beijing Zhongshan Jinqiao, China) staining were applied. Five high-power (×200) views were randomly selected for each section and scores estimated based on stain intensity and the percentage of positive cells.

### 2.3 Statistical analysis

Statistical analysis was conducted using SPSS 22.0 and GraphPad Prism 7.0. The measurement data were expressed as mean value ± SEM, while counting data were analyzed using the χ^2^ test and Fisher exact probability method. Spearman’s correlation coefficient was calculated for correlation analysis. Results were considered statistically significant when *P* < 0.05.

## 3 Results

### 3.1 Bioinformatics analysis showed that Ezrin was highly expressed in esophageal cancer and interacts with YAP_1_/CTGF

**Figure 1:**
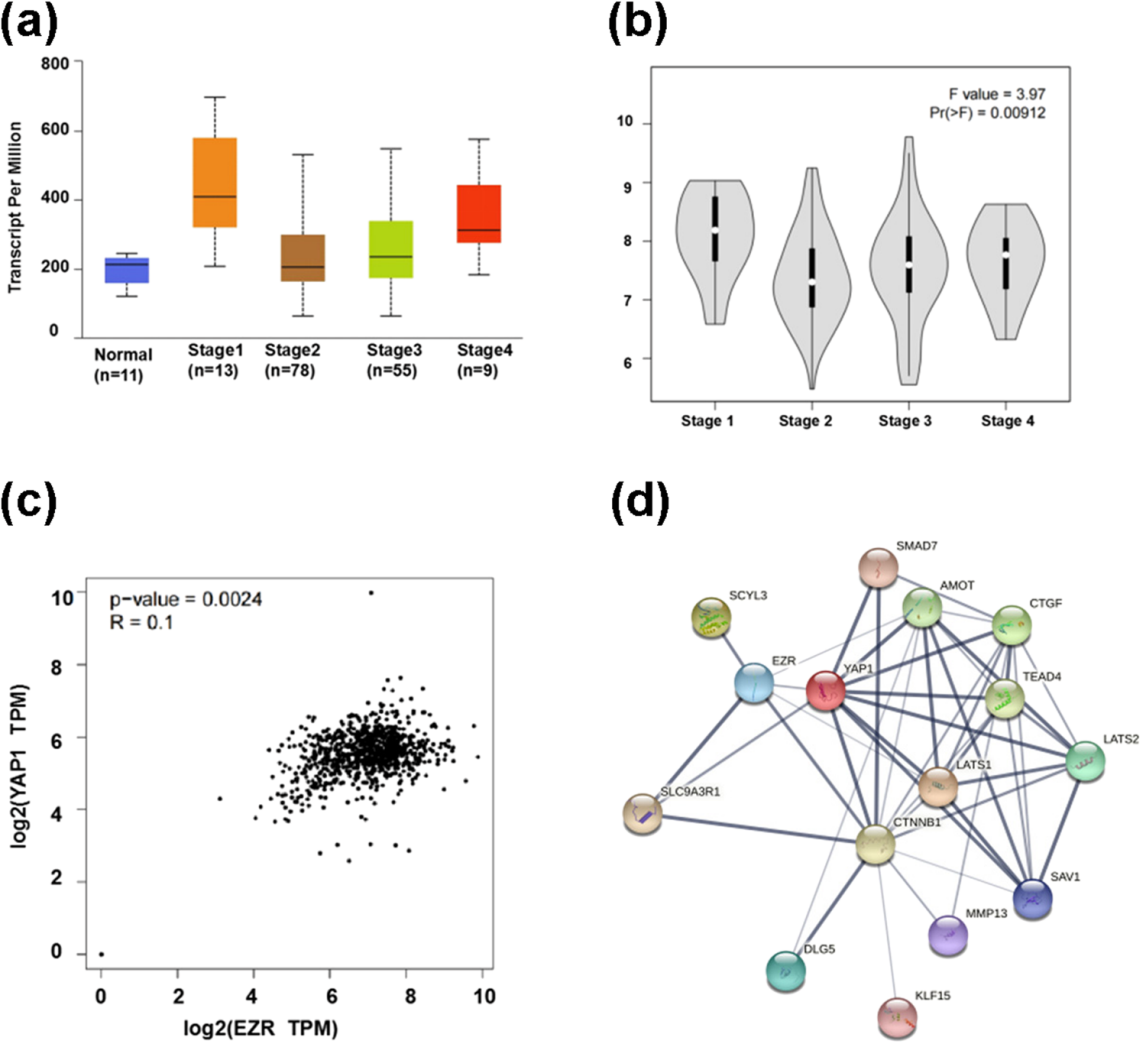
Correlation between Ezrin expression and tumor stage in data from patients with ESCC in the (a) UALCAN and (b) GEPIA databases. Analysis of the interaction between Ezrin and YAP_1_ using (c) UALCAN and (d) STRING.

We analyzed the relationship between differential Ezrin expression and ESCC cancer stage in patient data from the UALCAN database. Ezrin mRNA expression in stage I tumors was significantly higher than that in normal tissue, and Ezrin mRNA expression levels tended to increase in higher stage tumors (Figure 1a). We also used GEPIA data to further determine that the differences in Ezrin mRNA expression in patients with different clinical stages of ESCC were statistically significant (*P* = 0.009; Figure 1b). Moreover, analysis of data from the GEPIA database revealed a significant correlation between Ezrin and YAP_1_ levels in esophageal cancer tissue (*P* = 0.0024; *R* = 0.1) (Figure 1c). Searching for Ezrin and YAP_1_/CTGF in the STRING database indicated that there was an interaction between them (Figure 1d). Taken together, these data suggest that Ezrin and YAP_1_/CTGF may form a complex molecular network of interactions, which is potentially related to the carcinogenic mechanism. Thus, Ezrin likely plays an important role in esophageal cancer occurrence and development.

### 3.2 High expression and positive correlation between Ezrin and YAP_1_/CTGF in esophageal cancer tissue

**Figure 2:**
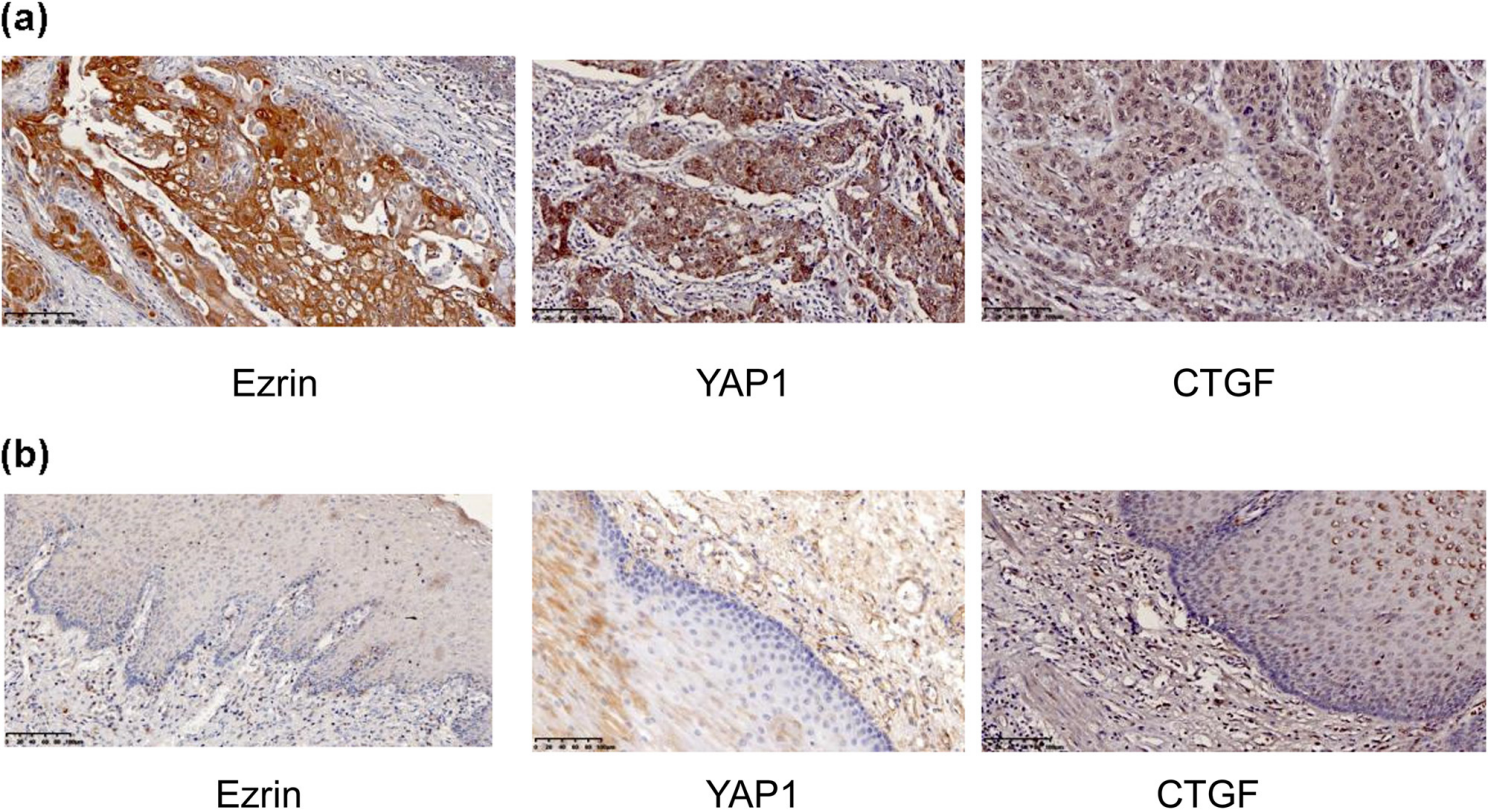
Expression levels of Ezrin, YAP_1_, and CTGF in ESCC (a) and adjacent (b) tissues (magnification, 200×).

Ezrin and YAP_1_/CTGF expression levels in esophageal cancer tissue were analyzed by immunohistochemistry (Figure 2). The positive expression rates of Ezrin, YAP_1_, and CTGF in cancer tissues were 86.7, 91.6, and 88.3%, while corresponding adjacent tissues showed low expression rates of 23.3, 11.7, and 18.3%, respectively. These data suggest that the positive expression rates of Ezrin and YAP_1_/CTGF were significantly lower in adjacent tissues than those in ESCC tissues (*P* < 0.001; Table 1). Spearman correlation analysis found a positive correlation between Ezrin and CTGF (*P* = 0.014, *r* = 0.316), Ezrin and YAP_1_ levels also showed significant and positive correlation (*P* < 0.001, *r* = 0.414, Table 2).

**Table 1:** Expression of Ezrin, YAP_1_, and CTGF in ESCC and adjacent tissues

Category		Pathological parameters/*N* (%)		*χ* ^*2*^ value	*P* value
		ESCC tissues	Adjacent tissues		
Ezrin	Positive	52 (86.7)	14 (23.3)	48.620	<0.001
	Negative	8 (13.3)	46 (76.7)		
YAP1	Positive	55 (91.6)	7 (11.7)	76.885	<0.001
	Negative	5 (3.4)	53 (88.3)		
CTGF	Positive	53 (88.3)	11 (18.3)	59.063	<0.001
	Negative	7 (11.7)	49 (81.7)		

*P* ＜ 0.05, the difference was statistically significant.

**Table 2:** Correlation between Ezrin and YAP_1_/ CTGF expression

Category		Ezrin		*r* value	*P* value
		Positive	Negative		
YAP1	Positive	50	5	0.414	<0.001
	Negative	2	3		
CTGF	Positive	48	5	0.316	0.014
	Negative	4	3		

*P*＜0.05, the difference was statistically significant.

### 3.3 Ezrin protein expression was significantly down-regulated in the sh-Ezrin group

**Figure 3:**
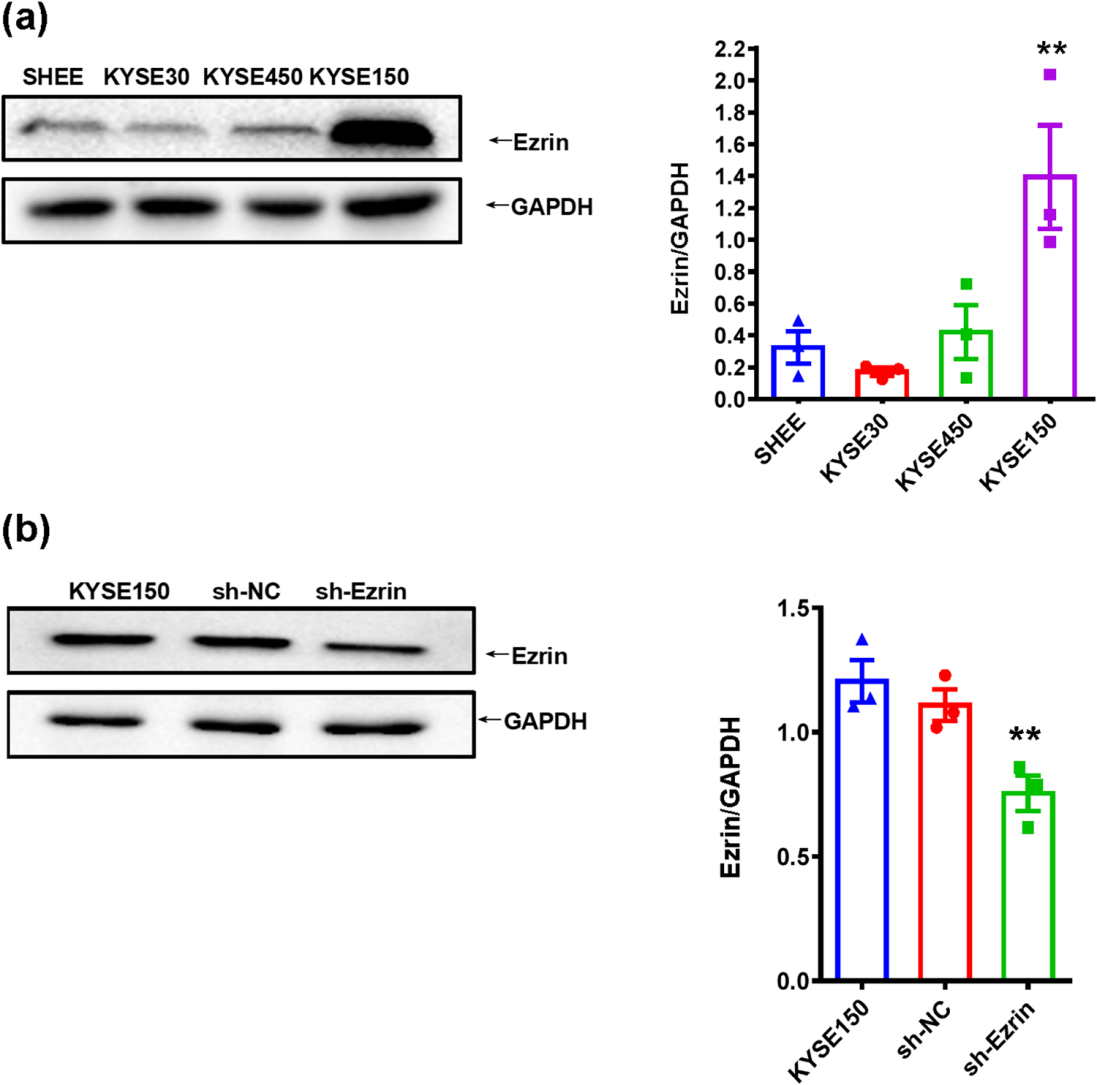
(a) Ezrin protein expression in esophageal epithelial cells and esophageal squamous cell cancer lines detected by western blot analysis. **P < 0.01 vs SHEE. (b) Western blot analysis of ezrin expression after lentivirus transfection. **P < 0.01 vs KYSE150.

Western blot analysis was conducted to detect Ezrin protein expression. The results showed that Ezrin was expressed in all three ESCC cell lines, and the expression was highest in KYSE150 cells compared with the normal esophageal squamous cell line, SHEE (*P* = 0.004, Figure 3a). Therefore, we chose KYSE150 for use in subsequent experiments.

Following lentivirus transfection, western blot analysis was conducted to analyze Ezrin expression at the protein level. Relative to KYSE150 cells in the control group, Ezrin protein expression in the sh-Ezrin group was significantly down-regulated (*P* = 0.005, Figure 3b).

### 3.4 Knockdown of Ezrin inhibited ESCC cell migration and invasion

**Figure 4:**
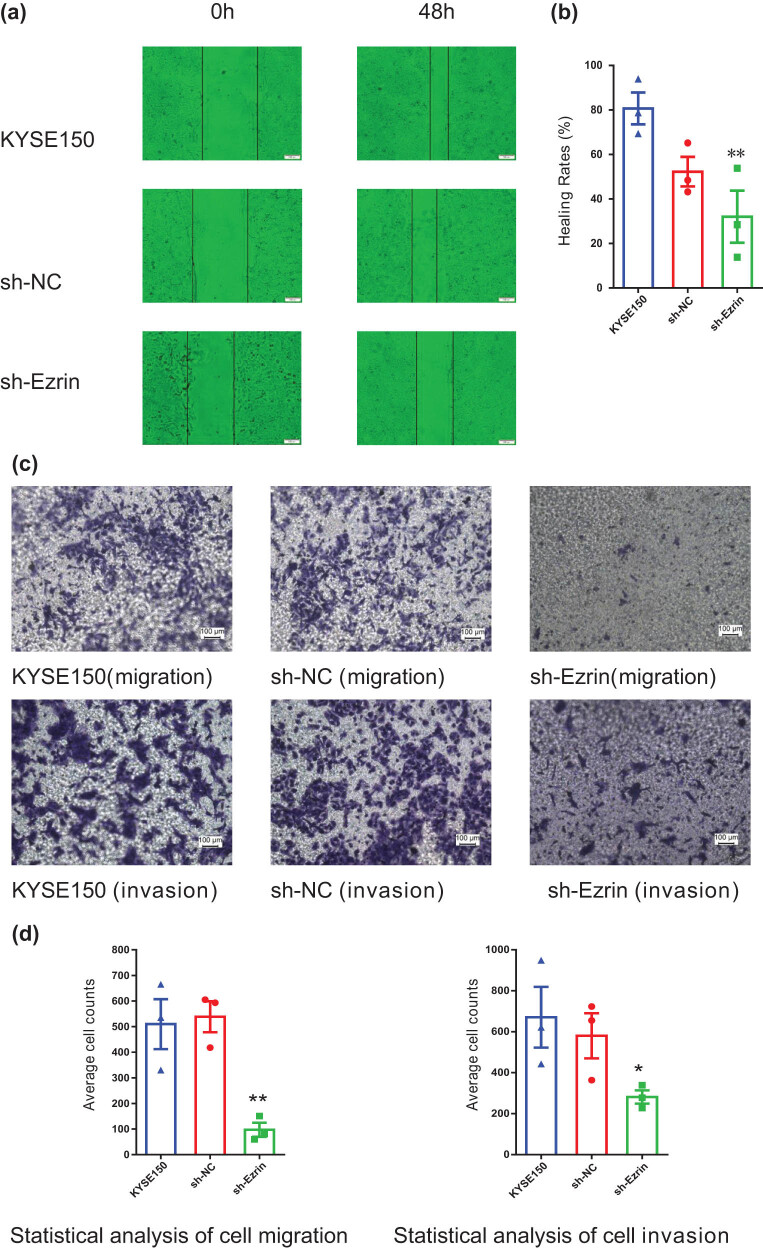
Knockdown of Ezrin inhibited ESCC cell migration and invasion. *P < 0.05, **P < 0.01 vs KYSE150. (a) and (b) Scratch healing test for analysis of ESCC cell healing after cell transfection (magnification, 100×). (c) and (d) Transwell assay evaluated ESCC migration and invasion after cell transfection.

Scratch test experiments revealed that KYSE150 cell migration was attenuated after Ezrin knockdown; the scratch healing rate of the KYSE150 group was 80.70 ± 7.16%, while that in the sh-Ezrin group was 32.04 ± 11.69%; hence the migration rate in the sh-Ezrin group was significantly lower than that of the KYSE150 control group after 48 h (Figure 4b, *P* = 0.008). This finding indicates that Ezrin knockdown can reduce tumor cell migration ability.

To further clarify whether Ezrin can inhibit ESCC cell migration and invasion, we conducted Transwell chamber assays to evaluate cell invasion and migration rates. The results showed that the number of migrated and invaded cells was significantly lower in the sh-Ezrin group than that in the KYSE150 group (Figure 4d, *P* < 0.05), suggesting that Ezrin can promote ESCC metastasis and invasion and may be a therapeutic target to prevent ESCC metastasis.

### 3.5 Ezrin promoted ESCC cell proliferation

**Figure 5:**
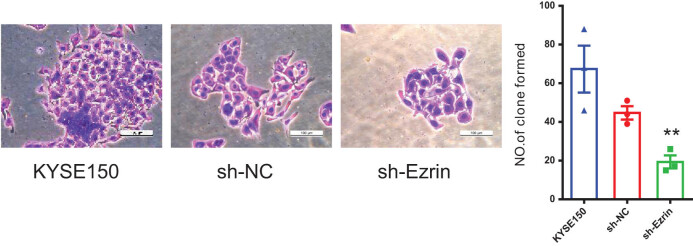
Plate cloning experiment to detect the number of colonies in the KYSE150 group, sh-NC group, and sh-Ezrin group. **P < 0.01 vs KYSE150.

Next the effects of Ezrin on cell colony forming ability were assessed using a plate cloning test. The number of cell clones in the KYSE150 control group was significantly and markedly higher than those in the sh-Ezrin group (*P* = 0.004), demonstrating that Ezrin can boost ESCC cell proliferation (Figure 5).

### 3.6 Ezrin promoted upregulation of YAP_1_ and CTGF protein levels

**Figure 6:**
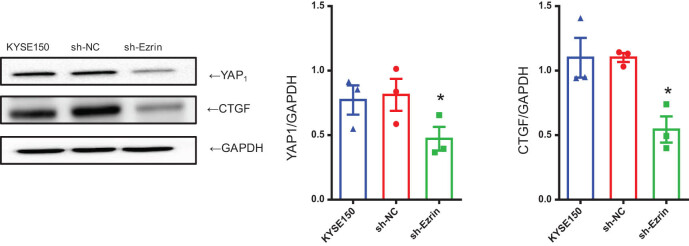
Western blot analysis showing YAP_1_ and CTGF protein levels in ESCC cells. *P < 0.05 vs KYSE150.

To further investigate the relationship between Ezrin and YAP_1_/CTGF expression, we analyzed YAP_1_ and CTGF protein levels in ESCC cells using western blots. YAP_1_ (*P* = 0.026) and CTGF (*P* = 0.011) protein levels were significantly lower in the sh-Ezrin group than those in the KYSE150 control group (Figure 6), indicating that Ezrin expression is positively correlated with YAP_1_ and CTGF activity. Therefore, Ezrin may be involved in the enhancement of YAP_1_ and CTGF protein expression during ESCC development.

## 4 Discussion

ESCC is among the most aggressive malignant tumors, and its incidence rate is increasing annually. Although modern medicine has made great progress in chemotherapy, radiotherapy, and molecular targeted therapy in recent years, most cases of ESCC are diagnosed at a late stage of the disease. Due to rapid metastasis and poor prognosis, early accurate diagnosis is of great significance to improve the overall survival rate of patients with ESCC. Ezrin protein is found in almost all human tissue cells and involved in the regulation of tumor cell adhesion, motility, signal transduction, and metastasis. Further, Ezrin is up-regulated in various cancers and associated with poor prognosis [20,21,22]. Additionally, the Ezrin gene enhancer exhibits enhanced transcriptional activity in pancreatic cancer [23]. Ezrin is mainly distributed in lung cancer tissues and metastases, and its expression is significantly correlated with the degree of lymphatic metastasis, malignant phenotype, and advanced TNM stage in patients with lung cancer, while downregulating Ezrin protein can reverse lung cancer malignant behavior [24]. Imaging characteristics of children with osteosarcoma combined with KI67 and Ezrin gene expression were used to establish a model that can accurately predict cell metastasis in this context [25]. Overall, the available evidence suggests that regulation of Ezrin gene transcription may exhibit similar characteristics in different types of tumor cells, and that Ezrin has potential as a valuable prognostic biomarker and biotherapeutic target in tumors.

Bioinformatics analysis confirmed that Ezrin is highly expressed in ESCC, and we identified a significant difference in Ezrin expression among esophageal cancer at different clinical stages. Immunohistochemical analysis showed that the positive expression rates of Ezrin, YAP_1_, and CTGF in paracancer tissues were 23.3, 11.7, and 18.3%, while those in ESCC were 86.7, 91.6, and 88.3%, respectively, displaying significant differences. We speculated that the abnormally high expression of Ezrin, YAP_1_, and CTGF in ESCC may be closely related to ESCC occurrence and development. By conducting a literature review, we found that Ezrin is significantly up-regulated in various tumors, including esophageal cancer, but the specific pathways and mechanisms involved remain unclear. Therefore, we used western blot analysis to detect Ezrin expression in different esophageal squamous cell lines, and selected the KYSE150 line for further study. Ezrin knockdown efficiency was verified by western blot. Subsequently, Functional cell culture experiments were conducted to observe the impact of Ezrin silencing on ESCC cell proliferation, migration, and invasion. Further, we conducted molecular analysis to observe the impact of Ezrin knockdown on core factors of the Hippo signaling pathway.

Invasion and metastasis are major biological characteristics of malignant tumors, and the main causes of clinical treatment failure and death. Ezrin expression is greatly increased in breast cancer cells and involved in regulating breast cancer cell proliferation, apoptosis, adhesion, invasion, metastasis, and angiogenesis [7]. Abnormal expression of Ezrin protein can confer high metastatic activity on tumor cells, affect and participate in multiple processes involved in tumor metastasis, and may even be a factor determining tumor metastasis [26]. In this study, cell proliferation and migration were measured using the plate cloning and cell scratch methods, respectively. We found that Ezrin knockdown inhibited KYSE150 cell proliferation and migration, consistent with the findings of a study where Ezrin gene knockout decreased proliferation and migration of pancreatic cancer cells [27]. Additionally, we evaluated the impact of Ezrin knockdown on KYSE150 cell invasion and migration ability using Transwell assays, and found that Ezrin knockdown significantly reduced KYSE150 cell invasion and migration ability.

The classical Hippo signaling pathway mainly regulates cell proliferation and death by inhibiting the function of YAP, thereby maintaining tissue and organ homeostasis [28]. YAP is involved in various fundamental biological processes, including cell proliferation, apoptosis, and drug resistance, and has important roles in tumor occurrence and development [29]. Ezrin and YAP_1_ are highly expressed in tumor cells, and Ezrin can regulate YAP_1_ expression, thereby promoting cancer occurrence and development [30]. CTGF, a cysteine-rich polypeptide, has significant roles in biological mechanisms including angiogenesis, cell proliferation, and cell movement; it is a downstream gene of YAP_1_, and its expression directly affects the YAP_1_ pathway, which is important in cancer progression. CTGF is important in progression of various types of tumors, where it can both promote and inhibit tumor progression. High CTGF expression in cells can promote tumor cell malignant progression [31], while CTGF is down-regulated and negatively correlated with invasive ability in colorectal cancer tissues and cells [32]. In this study, to confirm the regulatory effect of Ezrin on the YAP_1_/CTGF signaling pathway in ESCC cells after Ezrin was knocked down, YAP_1_ and CTGF protein levels were analyzed by western blot. The results showed that, after Ezrin knockdown, YAP_1_ and CTGF protein levels were significantly down-regulated. We infer from this result that Ezrin may promote ESCC occurrence and development, and its impact on ESCC malignant behavior may occur via up-regulation of YAP_1_ and CTGF expression in the Hippo pathway; however, our findings cannot fully and accurately explain the mechanism underlying the interaction between Ezrin and other core components of the Hippo pathway, which will be a major aspect of follow-up research on this subject. Comprehensive and in-depth analysis of Ezrin will provide new information regarding the basic mechanisms underlying ESCC activity. From a therapeutic perspective, our findings could contribute to the identification of new drug targets for ESCC prevention and treatment.

## 5 Conclusion

Ezrin can improve ESCC cell proliferation, migration rate, and invasion ability, indicating that Ezrin may be an important factor in ESCC occurrence and development, thus providing new insights that could inform ESCC treatment. In addition, Ezrin protein expression was positively correlated with YAP_1_/CTGF protein levels, suggesting that Ezrin may be involved in ESCC progression through the YAP_1_/CTGF signaling pathway. Overall, Ezrin inhibition can delay ESCC progression.
